# Digital Health Technologies: Learnings and Perspectives From a Patient Engagement Stakeholder Expectations Matrix Study

**DOI:** 10.2196/81463

**Published:** 2025-12-09

**Authors:** Leanne West, Derick Mitchell, Stuart D Faulkner, Birgit Bauer, Nicholas Brooke, Elizabeth Priest

**Affiliations:** 1 Georgia Institute of Technology Atlanta, GA United States; 2 Patient Focused Medicines Development (PFMD) Brussels Belgium; 3 University of Oxford Oxford United Kingdom; 4 Data Saves Lives Deutschland Munich Germany; 5 The Synergist Brussels Belgium

**Keywords:** digital health, patient engagement, stakeholder perspectives, health technology, cocreation, digital health literacy, patient-centered design, health data governance, multistakeholder collaboration, artificial intelligence in health care, system-level impact, digital therapeutics

## Abstract

As digital health technologies become increasingly integrated into health care systems worldwide, there is growing recognition that their full potential can be realized only when development is rooted in patient engagement (PE). Despite its proven value in clinical research and health care delivery, PE remains insufficiently embedded in digital health design and implementation. This perspective paper explores the current state of PE in digital health through findings from the Stakeholder Expectations Matrix program developed by Patient Focused Medicines Development. Drawing from 37 in-depth interviews across 6 key stakeholder groups, complemented by insights gathered during a multisession cocreation track at the Patient Engagement Open Forum, this paper highlights differing perspectives on digital health, the barriers to meaningful engagement, and the fragmented nature of data governance and technology adoption. Findings point not only to significant gaps in shared understanding, infrastructure, and policy but also to clear opportunities for collaboration, including early recommendations for building a more inclusive and patient-centered digital health ecosystem, one that supports sustainable innovation, trust, and systemwide impact.

## Introduction

Digital health technologies have become an essential tool to strengthen health systems and improve global health outcomes, offering tangible benefits to end users as well as economic value [1,2]. These have been reflected in an increased number of studies showcasing that digital health tools can significantly improve health outcomes, for example, improving adherence to evidence-based therapies [3].

Digital health can encompass a wide range of technologies and applications, from big data analytics to smart wearables to patient portals. For this paper, we consider digital health to include those technologies that broadly support the patient perspective and embed the lived experience. These include, but are not limited to, information for patients (ie, consumer information, wearables, and tracking tools), delivery of care for patients (ie, medical devices, artificial intelligence [AI] algorithms, and digital therapeutics), and obtaining support for patients (ie, electronic health records and tools that support communication between patients and health care professionals; Textbox 1, [4]).

**Categorization of digital health from the patient’s perspective: insights from the Patient Engagement Open Forum Session, June 13, 2023 [4].**

Information for patients

Purely informational services, applications, and solutions that do not require clinical evidence. This category includes resources used for tracking, digital health literacy, training, and prevention to improve health, including:

- User-facing technologies that do not need clinical evidence
- Consumer health information
- Tracking app + data collection tool for general health and wellness tracking
- Digital trainings and capability-building resources
- Decision support software

Delivery of care for patients

Any digital technology claiming to treat, manage, or improve a condition and support health care delivery. This category requires clinical evidence and regulatory oversight or approval, including:

- Medical devices, including implantables, monitoring devices, and devices for robotic-enabled surgeries
- Personalized therapeutics, such as digital diagnostics and biomarkers
- Clinician support, including remote patient monitoring
- Digital therapeutics: software that delivers a therapeutic intervention, supporting medical claims to treat, manage, or improve a condition (DTx)

Obtaining support for patients

Anything that helps a patient manage their own condition, including administrative and management tools, including:

- Digital software or platforms that support communication between health care professional and patient, including tools to support adherence, medication reminders, and access to test results and feedback
- Electronic health records
- Health care professional or institutional patient portals
- Interactive health care condition–specific apps that let you manage your own condition without medical claims and not requiring regulatory approval

Other technologies supporting digital health: Blockchain, artificial intelligence, machine learning, big data, interoperability, and Internet of Things

Elements supporting the patient perspective and embedding lived experience: Early and meaningful patient engagement; patient advocates, organizations, and registries; patient experience data (including real-world evidence); patient-supported data governance (data sharing and privacy); research with patients; and partnership with multistakeholder organizations working in digital literacy

However, the rapidly evolving landscape, especially with the rise of AI-assisted technologies, can be challenging to navigate. Stakeholders may struggle to understand the extent of what digital health encompasses, as well as the value of these technologies to patients across the spectrum of research and care. Patients, in particular, often report feeling that they lack the means to discuss and engage with digital health and are uncertain about where to learn about it, even when technologies are directly intended for their benefit (eg, virtual health visits) [5].

Such challenges often contribute to low adoption and missed opportunities for digital health to deliver on its potential [6,7]. In addition, the siloed development of digital health technology has resulted in many overlapping midquality technologies that encounter problems with their uptake, adherence, interoperability, and sustainability.

In this paper, we propose patient engagement (PE), defined here as “working with patients as partners in decision making to identify and articulate needs [and] design and co-create solutions,” as a strategy to overcome these challenges. While PE is increasingly recognized in many aspects of health care, including clinical trial programs [8,9] and across the medicines lifecycle, and expectations for integrating PE are reasonably well understood [10] and increasingly supported by frameworks, tools, and best practices [11-13], it remains insufficiently integrated in digital health. This issue is exacerbated by the fact that health care stakeholders lack a shared language, making it difficult to find alignment on the terms around PE and digital health.

Ideally, PE should be considered in all aspects of digital health including ideation, design, development, and implementation. Although there are isolated instances, it is not standard practice across digital health and software industries [14]. Thus, there remains a need to better understand stakeholder expectations around PE in digital health more broadly that can inform the development of multistakeholder frameworks on how to develop digital health technologies with patients at the outset or the earliest possible stage.

To this end, Patient Focused Medicines Development (PFMD) [15] has initiated efforts to operationalize and advance PE within digital health. Since 2021, PFMD has convened a community of early adopters in multiple Patient Engagement Open Forum (PEOF) [16] sessions, issued calls [5] for meaningful and sustained engagement, and cocreated solutions that are more aligned with the patient community’s needs. As part of this work, PFMD has supported a program to develop a Stakeholder Expectations Matrix (SEM) in digital health. This program tested hypotheses and expectations through a series of interviews with stakeholders to assess alignment, identify key barriers and opportunities, and provide a foundation for future multistakeholder frameworks that shift the thinking toward PE as an integral component of digital health development and deployment.

## Aims

This paper presents findings from the SEM program, together with qualitative insights from PEOF sessions (2021-2025) [16], to explore how different stakeholders approach PE in digital health and inform the authors’ perspectives on future opportunities.

Specifically, this work aimed to (1) develop a clear matrix encompassing all views from major stakeholders involved in the digital health ecosystem, (2) generate preliminary recommendations for aligning digital health solutions with broader health system goals and improved patient experience, and (3) set the conditions for further consensus on framework development of best practice.

## Approach

Data gathering followed a 2-stage approach: (1) semistructured interviews to inform the development of the SEM followed by (2) triangulation of ideas and recommendations through multiple open forum (PEOF) sessions. In stage 1, Monmouth Partners, an independent health care advisor, conducted 37 interviews lasting 40-60 minutes, with stakeholders from around the world. Interviews were carried out by 2 members of the Monmouth project team, both experienced in qualitative research and stakeholder engagement. The study process comprised 4 stages: project design, identification of interviewees, completion of the interviews, and critical analysis.

**Figure 1:**
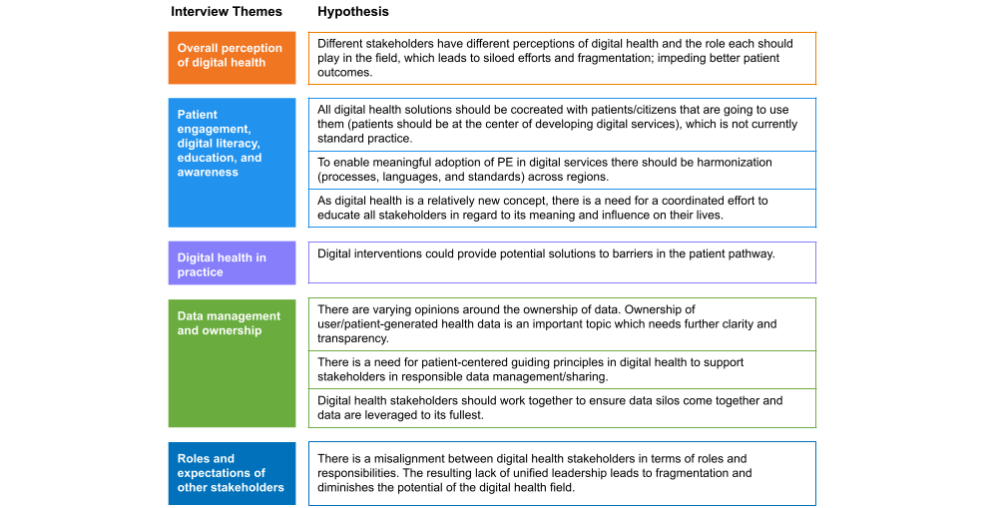
Core areas and related hypotheses of the SEM program interviews. PE: patient engagement

Interview topics were refined following a pilot phase and organized into five core areas: (1) overall perception of digital health; (2) PE, digital literacy, education and awareness; (3) digital health in practice; (4) data management and ownership; and (5) roles and expectations of other stakeholders, with an open category for “other considerations.” These themes, along with a set of hypotheses informed by previous PEOF sessions and experts’ consultations, are summarized in Figure 1.

Interviewees were selected using Quota and Snowball sampling to achieve a broad reach across geographies (focused on North America and Europe due to limitations on size and scope of the work program), job function, and experience. Participants were identified by approaching organizations and partners whose experience and expertise could add the greatest value and range of input. Inclusion criteria focused on ensuring that interviewees had direct experience or expertise in digital health, PE, or health care system innovation. Participants were approached if their role or organization could provide practical insights relevant to the study objectives and if they were available to participate in English language interviews. Organizations or individuals without active involvement in digital health or related PE activities were not included. Stakeholders were grouped into 6 categories: individual patients and patient organizations, industry, health care providers, technology developers, researchers, and policymakers. Table 1 outlines the group definitions and the number of interviewees by location. A semistructured interview guide was developed to explore stakeholder perspectives (Multimedia Appendix 1).

**Table 1:** Stakeholder group definitions, numbers, and locations of participant stakeholders.

Group	Definition	Number and location of interviewees
Patients, individuals, citizens, caregivers, patient advocates, or organizations	Individuals or organizations that represent the patient/citizen or patient advocacy perspective	8 (United States: n=2, United Kingdom: n=1, Romania: n=1, Netherlands: n=1, EU^a^-wide: n=1, Canada: n=1, and Belgium: n=1)
Industry (pharmaceutical, medical technology, and life sciences)	Organizations that are involved with the delivery or manufacturing of drugs, treatments, medical devices, data support or software developers, and diagnostic services	8 (Germany: n=3, Switzerland: n=2, Belgium: n=2, and United States: n=1)
Health care professionals	Health care professionals treating patients face-to-face or those who work within a health care provider from a digital perspective	3 (all in the United Kingdom)
Technology or digital companies	Digital solution developers with a focus on data, artificial intelligence practitioners or experts in health care, tech companies, health care strategy consultancies, or digital umbrella organizations	7 (United Kingdom; n=3, Spain: n=2, Switzerland: n=1, and Italy: n=1)
Researchers or academia	Researchers who develop clinical evidence or digital health researchers at research or academic institutions	6 (United Kingdom: n=4, United States: n=1, and Norway: n=1)
Policymakers, regulators, and payers	Organizations that support, create, monitor, or oversee health care policy or regulations, and organizations that pay for or recommend the commissioning of health care goods	5 (United Kingdom: n=2, United States: n=1, Netherlands: n=1, and Germany: n=1)

^a^EU: European Union.

The semistructured interviews’ data were collected between October 2022 and March 2023 by Monmouth project team. Interviews were recorded with prior informed consent from participants using Microsoft Teams and transcribed using Otter AI. The transcripts were reviewed and checked for accuracy against the recordings by the same team member who conducted the interview. In several cases, interviews were also transcribed live and later verified against the recordings to ensure accuracy. No other AI tools were used for data processing or analysis.

The Monmouth project team used grounded theory methods to conduct in-depth qualitative analysis of the results, involving systematic data collection, coding, categorization, and analysis to identify patterns and relationships. The resulting codes were iteratively grouped into themes, which were discussed with PFMD and refined through consensus to ensure consistency and reliability. PFMD hypotheses (summarized in Figure 1) were considered alongside the data to guide interpretation. Interview findings were then synthesized and complemented by the authors’ input to identify challenges, opportunities, and recommendations for PE in digital health.

It should be noted that there are some inherent limitations of this study. While a conscious effort was made to include a broad range of interviewees and to capture the views of each stakeholder group, individual participants cannot fully represent the breadth of views across their group. Notably, most participants were from Europe and North America, which may limit the applicability of findings to other regions. Nevertheless, the stakeholder groups encompassed a wide range of job roles, functions, and geographies, and individual stakeholders often held roles that straddled more than 1 category, contributing to a diverse and balanced dataset. In addition, it was not always possible to complete every interview question within the 40-60 minutes allocated; in such cases, time was focused on the areas of most relevance to the interviewee’s experience and expertise.

In stage 2, SEM initial findings were used to define the topics of PEOF cocreation sessions from 2023 to 2025 [16], hence providing an opportunity to test each finding and qualify it in light of the feedback received. These sessions also aimed to fill gaps from interview insights, particularly in areas such as PE in AI development, deployment, and human-centered innovation principles. This qualitative process informed the authors’ recommendations and possible future directions for consensus building.

## Data Management and Ethical Considerations

All interview recordings were conducted with explicit, informed consent from participants beforehand. Recordings were stored securely on Microsoft Teams and deleted automatically after 120 days, in line with Microsoft’s retention policy. All names and identifiable details were removed from transcripts and replaced with participant identifiers based on stakeholder group, job title, and territory. A separate participant key linking identifiers to individuals was stored securely by Monmouth project team. This key and all related data were subsequently deleted in accordance with their internal data retention policy and the agreed data management plan. All data processing was carried out in compliance with the UK GDPR (General Data Protection Regulation) and the Data Protection Act 2018. All participants provided informed consent for their anonymized statements to be used in publications, and all data have been handled in accordance with privacy and confidentiality standards. No new data were collected specifically for this paper. As the manuscript presents a viewpoint based on previously gathered and anonymized information, formal institutional review board approval was not required.

## SEM Findings

### Digital Health Is an “Umbrella Term”

All stakeholders acknowledged that digital health is an umbrella term covering a vast range of technologies, applications, and uses. As such, it often misses the purpose and method by which digital health is used. Interviewees reported that unclear definitions can cause unrealistic expectations and enthusiasm, as well as fear and prejudice around digital health. Variations in how stakeholders defined digital health were apparent both across and within groups. Interviewees also highlighted the complexities around the term “patient,” such as the question of whether an individual becomes a patient only when accessing health services.

For example, from a patient’s perspective, digital health may mean activities such as logging into a patient portal to access their electronic health records or using wearable sensors to monitor wellness. In contrast, payers may view digital health as provisioning health care to an individual via digital capabilities. Terminology can also vary across geographies and communities. These examples illustrate the lack of consistent terminology, which can result in misunderstandings, and highlight the difficulty of balancing inclusivity with clarity in definitions.

As one patient representative explained, “Everything that is digitized and relates to health could be considered ‘digital health’ at the widest definition. If you start to categorize, you might end up eliminating some that have potential benefits. On the other hand, if the definition is broad, people may understand the wrong thing about it and include things that are not relevant.”

A health care professional, on the other hand, noted that they would define digital health as “Any medium that allows a patient to access an experience of health that is not in a face-to-face setting.”

Similarly to what patient representatives highlighted, an expert from the tech/digital industry emphasized, “I think it’s context-based. If I am a software developer, I have one definition of digital health. If I am an individual patient who's sitting in front of my healthcare practitioner, I have a definition of what digital health is for me. If I'm on the other side of the desk than what digital health is, to me, it is something different. There will never be one unifying definition.”

A payer further warned that unclear definitions could set unrealistic expectations: “If digital health is poorly defined, people make it what they want it to be...And then later, down the road, everyone has to start thinking, why did it not meet those original expectations?”

With the objective of addressing this fragmentation in understanding, during the PEOF session held in June 2023, participants explored what stakeholders in digital health expect and need from each other [4]. The main outcome was that patients would like to see digital health classified in 3 ways: information resource, delivery of care, and self-management support (Textbox 1). These classifications may be one way for digital health stakeholders to structure engagement approaches, manage expectations, minimize misunderstandings, and improve patients’ comfort levels with these technologies.

### PE in Digital Health Should Center on the Patient

While all stakeholder groups agreed that PE is critical for the success of digital health technologies in solutions intended to support patients, there was a consensus that the level of meaningful PE in digital health is currently substandard. Specifically, interviewees from both the pharmaceutical and medical device industries noted that the shift in recent years of the “customer” focus from the prescriber to the patient is not as prevalent in digital health as in other areas, such as clinical research and regulatory approvals. As a consequence, the patient voice is often left out of early discussions in new digital health programs. In addition, interviewees underlined that digital health may have more than 1 end user (eg, an AI algorithm will affect the patient, but the “user” is the clinician).

### Recommendations and Suggestions

Several strategies are suggested to help boost PE in digital health, including unified methods, guidelines, or frameworks developed with patients for PE; forums for sharing experience and examples of good practice; clear regulatory guidance to address perceived ambiguity around requirements of PE across industry or technology; and education and awareness of the impact and benefits of PE in digital health. Another identified strategy is to ensure that all stakeholders improve their health literacy, including data and digital literacy, to empower patients and their advocates with the appropriate knowledge base to partake in PE activities. Interviewees also cited examples of what they perceived to be good PE in digital health (Table 2, including other examples cited during PEOF sessions).

**Table 2:** Cited examples of good patient engagement in digital health during the Stakeholder Expectations Matrix study and Patient Engagement Open Forum sessions.

Initiative	Description
Data Saves Lives [17]	A Europe-wide, multistakeholder initiative to raise wider patient and public awareness about the importance of health data, improving understanding of how it is used and establishing a trusted environment for multistakeholder dialogue about responsible use and good practices. This initiative includes the establishment of small pilot user advisory groups and patient advisory boards to provide feedback from the outset of a project or initiative and then grows with the program to educate throughout the process.
Patient Expert Center [18]	A Belgium-based, patient-led initiative that designs processes with industry and the health care system to ensure that all companies have the same processes to interact with patients more systematically and solidly than in the past.
Canada’s Drug Agency [19]	A Canadian national agency that involves patients in multiple aspects of their assessments of medical procedures, devices, and drugs. Canada’s Drug Agency signposts via their website the different ways in which patients can be involved in digital health.
IMI-Facilitate [20]	A 4-year IMI^a^ project to create a framework for clinical trial data to be returned to both clinical trial participants and their health care professionals.
The Public Engagement in Data Research Initiative [21]	A UK-based initiative that collaborates to improve public engagement practices in data and statistics by coproducing projects and engagement tools with the public (eg, Good Practice Standards).
LupusGPT [22]	A patient-driven, multilingual initiative, developed rapidly in a collaborative, multistakeholder process, with input from lupus patients, specialists, and industry partners. Regular updates continue to be incorporated based on user feedback, ensuring that the tool evolves to meet patient needs.
Parkinson’s UK Tech Guide [23]	A patient group initiative designed to increase the number of people with Parkinson disease using appropriate devices and apps that can positively impact their quality of life. Key features include “lived experience” reviews and a dedicated group of individuals with Parkinson disease and family members guiding the project’s direction and ensuring accountability.
EDiHTA [24]	A 4-year Horizon Europe project to create the first flexible, inclusive, validated, and ready-for-use European HTA^b^ framework reaching TRL^c^ 6-7, allowing the assessment of different digital health technologies (eg, telemedicine, mApps, and AI^d^) at different TRLs, territorial levels (national, regional, and local), and perspectives (eg, payer, society, and hospital). All relevant stakeholders, including the patient community, will contribute to its design, development, and validation.

^a^IMI: Innovative Medicines Initiative.

^b^HTA: Health Technology Assessment.

^c^TRL: Technology Readiness Level.

^d^AI: artificial intelligence.

### The Digital Health Marketplace Is Overwhelmed With Multiple Options

Interviewees perceived the use of digital health technologies by end users to be integral to health care in the present and future as part of the wider system. Multiple stakeholders stressed the need for clearer guidance to help patients identify clinically endorsed solutions, as the marketplace is currently overwhelmed with multiple options in the same areas. In addition, interviewees expressed concerns about privacy and data protection, emphasizing the need to support patients in leveraging only trusted solutions.

They also noted that, while individuals are generating vast amounts of health data, much of it remains siloed and underused due to a lack of interoperability and common oversight, ultimately hindering meaningful digital transformation. Finally, prioritization of digital health technologies in practice may vary, in part, by geography, depending on specific local pressures (eg, access to broadband, access to primary care, and lack of resources in rural areas, including shortage of specialists).

### Data Governance and Management Is a Complex Area in Digital Health

Ownership of health data was recognized as a complex area in digital health, with data owned, stored, and managed by different private or public entities across jurisdictions. This fragmented governance contrasts with the growing emphasis on patients’ rights to own their own health data, adding further complexity. For some interviewees, the concept of consent was a central concern. Specifically, some patients voiced concerns about hospitals using their bedside data for algorithm development, highlighting ongoing ethical and transparency challenges. Therefore, the need for greater transparency about what individuals are consenting to, particularly regarding secondary data use, and the risk that overly rigid consent processes may hinder research and data sharing.

This issue ties into the broader concept of informed consent in health care, a general challenge further amplified in the digital health landscape. Key areas to explore include the duration of consent and the limits to usage, especially given the evolving nature of digital health technologies and data-sharing practices. Interviewees also underscored the absence of systems and guidelines to support sufficiently agile processes around consent.

### Various Stakeholders Have Different Expectations and Definitions of Their Roles

While stakeholders generally agreed on the importance and benefits of PE in digital health, distinct expectations and roles were articulated for each group. Multimedia Appendix 2 summarizes the core roles, responsibilities, and potential actions defined by each stakeholder group, as well as the roles, responsibilities, and potential actions of the other groups, in the development and maturation of PE in digital health. Multimedia Appendix 3 summarizes key quotes from individual stakeholders to support their claims.

The stakeholder groups generally agreed that patients and patient organizations play key roles in engaging with the development and implementation of digital health, although there were variations in the level of engagement that stakeholders cited as desirable. While patients perceived their role as “defining the needs, gaps, and barriers in the pathway and the metrics that should be measured,” industry stakeholders expanded this role in being “effective partners in development” and that patients should “advocate harder to ensure General Data Protection Regulation (GDPR) is not a barrier to data sharing.”

Patient organizations saw their role as “acting as the trusted voices for patients” and “providing the collective intelligence of a disease,” while research or academia stakeholders emphasized that patient organizations could be part of a wider effort to provide education for patients on how to engage with digital health. Digital/tech company stakeholders felt that patient organizations should also “hold people accountable and be ethically-oriented to serve patient interests.” Industry stakeholders added that “while patient organizations are the link between individuals and developers, in order to reach a wider non-patient audience, the media (broadcast, journalism-based, and social media) and other stakeholders need to be involved.”

## Insights and Observations: Challenges to PE in Digital Health

Three key challenges to PE in digital health emerged from the interviews.

1. *Meaningful PE is suboptimal in digital health*: Historically, patients have been rarely involved in the early stages of digital health product development, which may result in poor adoption of and adherence to the final product [6,7]. For example, in the field of medical devices, the patient is not seen as the decision maker and is therefore left out of critical decisions, such as the governance and development of the product. Instead, patients are consulted only on superficial details related to the device, such as the design of manuals or visual features.

As one industry representative observed, “So often in my experience, when it comes to patient engagement, digital health is more about the adaptation of a concept or an idea rather than the real co-creation.” Similarly, a tech expert highlighted, “My perception is that in digital health, the applications are very much shaped by scientists, data scientists, medical professionals, but hardly ever by the patients and how they want their data to be used. There is a quite a big disparity between what the scientific community thinks data can bring by being analyzed, and what patients actually need on a daily basis.”

An academic representative contextualized some of the fears on the private sector side: “As much as we talk about patient centricity, for the pharma industry there's a big fear about regulation and compliance and not interacting directly with patients.”

2. *Patients are not considered as partners*: Patients perceive that their point of view is underrepresented due to digital companies not sufficiently recognizing the value of patient insight and experiences. “We absolutely see that there's increasing consideration for patient engagement and patient experience data within the regulatory and the Health Technology Assessment community,” explained one patient advocate, “but we're not there yet. There still needs to be more open, transparent communication with that.”

As digital health evolves, there is continuous work to be done around digital health literacy (including data literacy), as well as education and awareness of the benefits, expectations, and regulations around patient and public involvement with digital and device stakeholders. Sometimes, even with user-friendly digital health solutions, policies of the payers or providers can restrict the effectiveness of the solution (eg, whether the payer or provider can legally give any useful advice through the patient portal, or whether there are set answers that they must stick to). To address this, digital solutions must prioritize patient needs and patient user requirements.

3. *Trust challenge in data usage*: Finally, the third key challenge revolves around trust issues over the use of personal data in digital health technologies. Data privacy and access remain sensitive topics for patients. The variety of platforms that require input of personal information from different providers or services can be overwhelming, especially when potential connections between sites are unclear or nonexistent.

Users may perceive that their health data are being used by companies for profit, and they may also express concerns about the privacy of their data and potential consequences of employers, payers, or even the government having access to their health information [25]. These concerns can create distrust, highlighting the need for transparency so that patients can understand how their information is being used and by whom. This lack of transparency and clarity around data use, combined with uncertainty about where patient data flow and who benefits from them, leads to a perceived imbalance of power and fuels mistrust in digital health ecosystems.

The potential misuse of patient information spans across different dimensions. For example, one patient questioned corporate motives: “When you think about some of the private companies that are using the data, is that for the health of society? Or is it that companies are doing it to be able to make some profit and generate some revenue from it?” On the other hand, another patient commented on the paradox of privacy: “I am fascinated with this whole thing of personal health data privacy, and people not wanting anybody to see it or use it. And then we flip over, and we post it on Facebook.”

In addition to privacy concerns, some stakeholders raised issues around emerging technologies such as AI. A policymaker warned, “I am increasingly worried that everybody's jumping on artificial intelligence as a solution in healthcare.” This skepticism reflects broader apprehensions about the integration of advanced technologies in health care without adequate understanding or oversight. Similarly, it underscores the specificities of trust in digital health, especially in relation to AI, where both the volume of data and the opaqueness of algorithms amplify existing insecurities.

**Figure 2:**
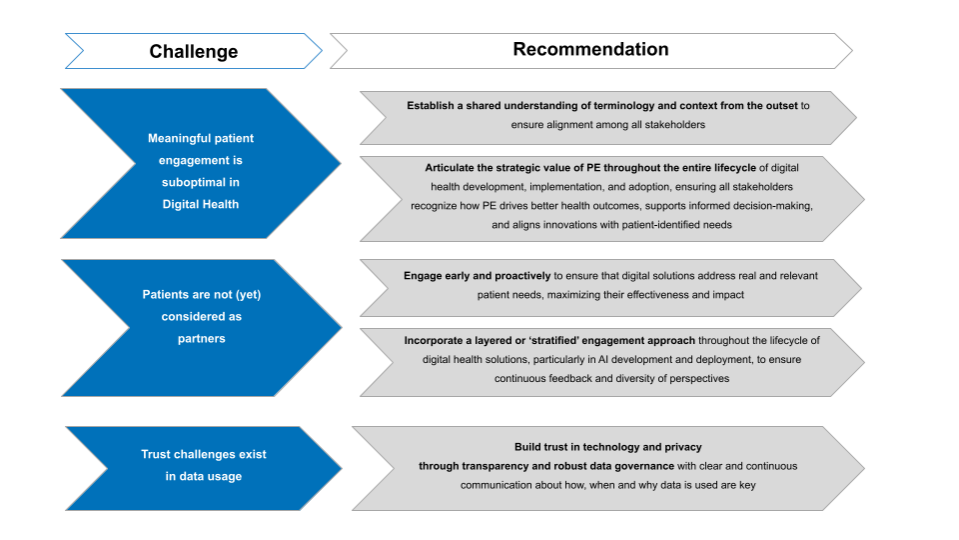
Challenges identified in the stakeholder interviews and authors’ recommendations. PE: patient engagement.

As discussed during PEOF 2025 [26], addressing these underlying tensions will be critical to building and maintaining trust in digital health, particularly as systems evolve to collect, connect, and interpret increasingly complex types of data. Our recommendations for addressing these challenges and improving PE in digital health are summarized in Figure 2.

## Authors’ Recommendations for PE in Digital Health

### Overview

To ensure meaningful PE in digital health development, we recommend that developers and deployers do the following:

1. Establish a shared understanding of terminology and context from the outset to ensure alignment among all stakeholders. This alignment requires moving beyond a “technology push” perspective to adopt a “health-outcomes-first” mindset, a shift that ensures that digital solutions are guided by patient needs and perspectives rather than driven solely by technological innovation. Cocreation and multistakeholder collaboration are essential elements to aligning digital health innovations with patient-centered goals and values. Clear and consistent communication facilitates this process, fostering coherence and engagement across all parties.
2. Articulate the strategic value of PE throughout the entire lifecycle of digital health development, implementation, and adoption, ensuring that all stakeholders recognize how PE drives better health outcomes, supports informed decision-making, and aligns innovations with patient-identified needs. Identifying critical touchpoints where PE should be enhanced and optimized is key to demonstrating its impact on both the quality and the relevance of digital health solutions. Highlighting these benefits builds confidence among all stakeholders, including investors, who increasingly value solutions that directly address patient needs [27,28].
3. Engage early and proactively to ensure that digital solutions address real and relevant patient needs, maximizing their effectiveness and impact, which can be achieved by adopting and adapting established frameworks from other fields, such as drug development, to guide PE strategies in digital health. Co-designing strategies with patients and other stakeholders ensures solutions that are not only functional but also meaningful to the end users [29]. Such engagement not only fosters trust but also allows digital health innovations to bridge the gap between technical capabilities and real-world health care outcomes [30].
4. Incorporate a layered or “stratified” engagement approach
  throughout the lifecycle of digital health solutions, particularly in AI development and deployment, to ensure continuous feedback and diversity of perspectives. In practice, appoint a core group of patients to provide consistent input throughout the development process, while also involving different patients at specific phases based on the expertise and diversity needed at each stage. This layered approach enriches insights, reduces bias, and ensures that solutions are not only relevant but also inclusive.
5. Build trust through transparency and robust data governance
  to address the predominant concerns of all stakeholders, especially patients, toward digital solutions. Issues of data sharing, privacy, and security are particularly critical in digital health, where concerns about trust are often amplified. To overcome these concerns,
  clear and continuous communication about how, when, and why data are used
  is required. Patients should play an active role in designing data governance frameworks to ensure alignment with their expectations and priorities. Transparent practices should drive interactions to foster trust and encourage patient participation, supporting the development of digital solutions that meet both patient needs and ethical standards. Transparency is thus framed not just as an ethical imperative but as a practical enabler of patient participation and solution sustainability.
6. Recognize that one patient does not represent all patients. Including patient perspectives means engaging more than 1 patient to account for variability of experiences, preferences, and concerns that exist within any patient group.

These recommendations reflect both the coherence of PE principles across diverse contexts and the unique challenges posed by digital health. The first 3 recommendations underscore the consistency of expectations in PE, demonstrating that the core principles—shared terminology, the articulation of value, and early engagement—apply universally, including in digital health. These principles align with broader PE practices, providing a strong base for collaboration and innovation.

Our exploration reveals that the key difference is not in the principles themselves but in their practical application, underscoring the current failure to consistently translate these expectations into practice within digital health. Existing tools and guidance, such as PFMD’s PE road map in digital health [31], and similar road maps from medicines lifecycle development [32] can help operationalize these recommendations. By focusing on actionable steps, such as early involvement of patients in co-design, defining data governance, and establishing metrics of success with patients, the road map supports stakeholders to align digital health with patient needs, ultimately increasing adherence and sustainability of these technologies. The next 2 recommendations are particularly pertinent to AI-enabled health technologies. AI systems depend on large volumes of data and involve opaque decision-making processes that can amplify inequities or unintended harms if patients are not meaningfully involved [33].

Finally, understanding that patients are different and have different concerns, experiences, insights, and goals is important. One patient does not speak for all patients. It benefits the developers to obtain a wide array of patient perspectives to ensure that the developed solutions actually meet the needs and reduce the concerns of the patient group as a whole.

With patient organizations [34] and members of the public [35] increasingly expressing their desire for involvement in algorithm development since it will affect individual treatment and possibly health outcomes, the need for a multistakeholder approach where the patient and public voice is central is apparent. A layered engagement approach helps ensure ongoing, diverse input throughout the algorithm development lifecycle, enabling the identification of risks, the validation of assumptions, and the shaping of AI models to reflect patient values and lived realities.

Similarly, as highlighted in the SEM interviews, trust issues related to data remain a key concern overall and are particularly enhanced in the context of AI-based technology. Challenges related to data usage, privacy, and governance are central to digital health, making transparency paramount to building trust. The increasing focus on the link between data management and trust makes evident the specificities of digital health: while the foundational principles of PE remain consistent, the practical challenges in this space require tailored solutions to address its distinct nature.

A paradigm shift is therefore needed: by actively involving patients in the design, implementation, and evaluation of digital health solutions, and by addressing current implementation gaps and unique challenges, stakeholders can ensure that innovation leads to impactful, trusted, and sustainable outcomes that truly reflect patient needs and strengthen the broader health ecosystem.

### Unresolved Tensions

The evolution of digital health offers immense potential for improving health care outcomes, yet the SEM program reflections, along with insights gathered during PEOF sessions, surfaced several tensions that stakeholders must collectively address to ensure meaningful progress in PE:

- Patient-focused versus payer-focused solutions: Balancing the needs of individual patients with those of payors or providers and broader health system efficiency presents a fundamental challenge. Digital health must navigate this duality, ensuring that solutions serve both personal and systemic priorities.
- Patient-centered benefits versus health system efficiency: A closely related issue is the tension between prioritizing patient-centered improvements, such as accessibility, literacy, and outcomes, and addressing the overarching goal of health system sustainability. Achieving alignment between these priorities is crucial for long-term success.
- Established versus emerging PE paradigms: While pharmaceuticals and Medtech offer valuable insights into PE [36-38], digital health’s vast range from digital therapeutics to medical devices, as well as its reliance on real-time data and rapid innovation cycles, highlights the need for approaches that address its unique dynamics, and possibly tailored solutions to specific domains.
- Transparency versus conflicting data expectations: Stakeholders have different expectations about what qualifies as health data, what should be shared, and under what conditions. This lack of alignment contributes to uncertainty and erodes trust, especially when the boundaries between what constitutes personal, clinical, and commercially valuable data are not clearly communicated.
- Patient versus professional understanding of technology: Not every patient is technically savvy and may not understand how data are being used or the complexity of the different use cases of AI. Clear communication and education between stakeholders can alleviate misunderstanding and distrust.

### Potential Next Steps and Areas to Build Upon

Based on these recommendations and unresolved tensions, in order to ensure that digital health technologies are designed, implemented, and sustained with patient needs at the core, a number of key next steps and areas for stakeholders to focus on are proposed, including the following:

- Address policy gaps explicitly: Extend preexisting analyses of digital health policies [39] to identify gaps and opportunities for alignment of PE explicitly with digital health needs. A clear regulatory framework will improve trust, innovation, and patient-focused development.
- Balance stakeholder priorities: Leverage existing collaborative frameworks [40] and multistakeholder projects [41] to harmonize patient needs with health system efficiency goals around digital health care. Multistakeholder approaches can ensure that digital solutions are both impactful for individuals and sustainable for systems.
- Tailor engagement to digital health’s diversity: Recognize the diversity within digital health (from big data analytics to smart wearables to AI) and adapt engagement strategies to specific contexts. Early, layered PE across the lifecycle mitigates bias while ensuring that solutions remain relevant and effective.
- Focus on building trust: Strengthen transparency by including patients and citizens in data governance design and investing in digital health literacy for users. For technologies involving secondary uses of data, implement dynamic consent mechanisms for ongoing control over data use, reflecting evolving preferences and sensitivities, which not only addresses privacy concerns but also fosters confidence in the technologies themselves. For example, the learnings from responsible health data use initiatives [42,43] can be used to cocreate governance frameworks that reflect patient values such as transparency, control, and benefit-sharing [44].
- Advance toward a new social contract: In addition to more meaningful, sustainable PE, digital health must embrace broader social participation and build civil society capacities [45], aimed at empowering citizens to coshape health technologies and policies. Building a social contract grounded in shared responsibility and aligned with public interest is key to ensuring accountability and sustaining trust as health systems grow more complex and data-intensive.

### Final Reflection and Call to Action

The digital health sector stands at a pivotal moment. It offers transformational and permanent change across the life course of a patient’s journey through the health care system. Addressing these unresolved tensions now will shape its trajectory. Efforts that combine consensus on broad principles and best practices, complemented by the development of rigorous contextualized frameworks to support the colorful scope of digital health, are needed today. By fostering collaboration, promoting education, and prioritizing trust and transparency, these multistakeholder approaches can ensure that PE becomes a cornerstone of digital health innovation. The PEOF has proven to be a successful platform to explore some of these approaches across a very large number and range of partners and geographies and will continue to do so with other partners. As one patient advocate eloquently summarized: “Today, we have to beg to have a seat at the table. I think in 10 years from now, we will set up the table and ask our partners to come.” This shift will position digital health as a sector that prioritizes patients’ needs while advancing sustainable and impactful health care solutions.

## Appendix

Multimedia Appendix 1 Stakeholder Expectations Matrix in digital health interview guide.

Multimedia Appendix 2 Tables summarizing stakeholder expectations by groups.

Multimedia Appendix 3 Selected quotes from Stakeholder Expectations Matrix interviews.

## Abbreviations

AI: artificial intelligence
GDPR: General Data Protection Regulation
PE: patient engagement
PEOF: Patient Engagement Open Forum
PFMD: Patient Focused Medicines Development
SEM: Stakeholder Expectations Matrix

## Data Availability

The data supporting this work are not publicly available due to confidentiality agreements with interviewees, but anonymized excerpts may be available from the corresponding author upon reasonable request through a formal data request.
