# Supplementary material for: Digital Health Technologies: Learnings and Perspectives From a Patient Engagement Stakeholder Expectations Matrix Study
Source: J Med Internet Res. 2025 Dec 9;27:e81463. doi: 10.2196/81463 (PMC12728396; doi:10.2196/81463)
Supplement: Multimedia Appendix 1 [file jmir_v27i1e81463_app1.pdf]

This is a Multimedia Appendix to a full manuscript published in the J Med Internet Res. For full copyright and citation information, see http://dx.doi.org/10.2196/jmir.81463

PFMD's Stakeholder Expectations Matrix (SEM) Interview Guide

Key Themes Summary:

1. ***Digital Health- overall perception***
2. ***Patient engagement, digital health literacy, education, and awareness***
3. ***Digital Health in Practice***
4. ***Data Management/Data Ownership***
5. ***Roles and expectations of the other stakeholders (For ALL Stakeholders)***
6. ***What are other things that we should consider/what else do you think it's important to know?***

Questions for interviews

1. **Digital Health - Overall Perception**
    - What does Digital Health mean to you (OR what is your definition of digital health?
    - What does Patient Engagement in Digital Health mean to you?
    - How do you think Patient Engagement in Digital Health should be defined?
    - What is the area of digital health that your work focuses on? *(for example: data sharing and management, real-world data or patient experience data, communication, European commission projects, etc.)*
    - Do you think patient engagement brings value to digital health? If so, why?
2. **Patient engagement, digital health literacy, education, and awareness**
    - What can be done to improve digital health literacy across the board, and whose mission (which stakeholder) would it be to do so?
    - We've heard that communication around PE in digital health is a challenge, and that duplication occurs because great initiatives are being drowned out by all of the other noise in the field. How do you think this can be overcome?

3. Digital Health in Practice

- Have you ever been recommended a digital tool (device/app/etc.) as a solution for a health issue (including mental health issues) by a medical professional? If yes, which one and what has been your experience with it? **(If the answer is NO)** Have you used a digital tool for a health issue (including mental health issues) by yourself? What has been your experience with it?
- We've heard in previous PEOFs that standardization of a common language in digital health and patient engagement would be helpful. How do you think this could be accomplished?
- How can Patient Engagement (PE) good practices be implemented on a large scale and systematically in digital health?
- How do you keep patients at the center of developing digital services?

4. Data Management/Data Ownership

- Do you consider there is sufficient transparency around who (which stakeholders) owns the health data and how it can be utilized? If not, how can this be improved, and whose stakeholder job would it be do so?
- How do we prevent duplication of data and support data sharing?
- How can we take the lessons learned from PE in drug development and bring them to digital health?
- How can all stakeholders work together for better data management?
- Who (which stakeholder) should own the health data?
    1. Do you have a set of data governance guiding principles that you use to handle all health data you store? If yes, were these principles developed internally or with other stakeholders (such as patients or regulators)? Are they based on some existing principles used in the field (such as FAIR)?
    2. Do you have lay language & dynamic consent forms that health data donors sign before handing in their data?
    3. Do you believe patient and patient organizations can act as data fiduciaries / managing the data commons and providing analytics to all stakeholders?

5. Roles and expectations of the other stakeholders (For ALL Stakeholders)

(to help narrow the focus, each person should answer reflecting on their own role and scope in PE in digital)

- What is the role of your stakeholder group in ensuring patients are included in the design and decision-making of digital health solutions?
- What are you or your stakeholder group doing in overcoming challenges around data sharing?
- These are all the stakeholder groups that we have identified (list stakeholders).
    1. Of these, which do you currently work with?
    2. Which have you not worked with? Why not? Is it appropriate / would you like to / how would you benefit
    3. Are there any that are a priority stakeholder for you? Why or why not?
    4. Is a collaboration between these stakeholder groups for patient engagement in digital health effective? Please explain. What works well / examples of what doesn't work well.
    5. Do you think all stakeholders have equal responsibility in PE in digital health?
- What is something that you feel the other stakeholders could improve upon?
- Looking ahead, what should your stakeholder group do to thrive in the digital health environment?

6. What are other things that we should consider/what else do you think it's important to know?
