# Supplementary material for: Digital Health Technologies: Learnings and Perspectives From a Patient Engagement Stakeholder Expectations Matrix Study
Source: J Med Internet Res. 2025 Dec 9;27:e81463. doi: 10.2196/81463 (PMC12728396; doi:10.2196/81463)
Supplement: Multimedia Appendix 2 [file jmir_v27i1e81463_app2.pdf]

This is a Multimedia Appendix to a full manuscript published in the J Med Internet Res. For full copyright and citation information see http://dx.doi.org/10.2196/jmir.81463

Table 1. Stakeholder Expectations Snapshot. Summary of Interview Findings: Healthcare Professionals or healthcare providers

	Patients/ Individuals/ Advocates on PO	HCPs or healthcare providers	Researchers/ academia	Policymakers, regulators and payers	Industry	Tech/digital companies
HCPs or Healthcare Providers	Provide feedback on their experience, pain points in their pathway and needs<br><br>Want them to be engaged and activated in their own health<br><br>Engage communications about their health and the benefits of activation/dh<br><br>Adopt in the initiatives when they offer real benefit<br><br>Priority stakeholder	Big driver for a lot of internal solutions<br><br>Educating clinicians to understand that digital modalities help amplify them<br><br>Key and user for a lot of DH solutions<br><br>Provide experience and knowledge to develop tools	Be open to parameters like real world evidence, and broaden the terms of what is currently accepted data/evidence	Incentivize people through DH and gamification to engage in their health	Provider of a disease management program with a digital solution<br><br>Need to spend time education HCPs and end users of the benefits	Develop technology that genuinely supports the healthcare system and workforce

Table 2. Stakeholder Expectations Snapshot. Summary of Interview Findings: Research / Academia.

	Patients/ Individuals/ Advocates on PO	HCPs or healthcare providers	Researchers / academia	Policymakers, regulators and payers	Industry	Tech/digital companies
**Researchers/ academia**	Be a partner in the design, co-creation, implementation, and dissemination of Digital Health Solutions<br><br>POs could be part of a wide effort to provide education to patients about how to engage with DH (cannot do in isolation)	Utilise the technology. (That goes in hand with a lot of education)<br><br>Professional bodies can support upskilling<br><br>Think about the level of consolidation that would be helpful from industry to support patients more effectively.	Crucial players in implementation - 'Beta testers for the real world'<br><br>Support questions around what patient-collected health data is food/robust evidence in clinical setting<br><br>Include more social science in research around DH and impact/outcomes<br><br>Genuinely involve patients in research	Set data quality standards<br><br>Provide frameworks for how data should be utilised to inform drug approvals, clinical trial designs, policy decisions.<br><br>Consider including subjects like: consenting data for research/how to interact with healthcare data, and how it can be used to inform healthcare decisions, in the school curriculum<br><br>Government could host a web-based resource to explain terminology to the public around DH	Responsibility in making sure that DH solutions are addressed equitably and diversely<br><br>Use much plainer language<br><br>Make sure they are working within the correct frameworks/ or in a way that frameworks can accommodate them	Involve patients by default. Make sure solutions are user-friendly

Table 3. Stakeholder Expectations Snapshot. Summary of Interview Findings: Patients/ Individuals/ advocates or POs.

	Patients/ Individuals/ Advocates on PO	HCPs or healthcare providers	Researchers/ academia	Policymakers, regulators and payers	Industry	Tech/digital companies
Patients/ Individuals/ Advocates on PO	To define needs, gaps, barriers in the pathway, outcomes and the metrics that should be measured<br><br>At an individual level, embrace technology and learning. (need good guidance) to support their own health/care<br><br>POs to act as the trusted voices for patients<br>Advocate for the patient population as the 'custodians of the patient journey'.<br>Endorse resources and projects.<br>Educate members about PE in DH.<br>Provide the collective intelligence of a disease - both clinical and wider social or mental health aspects - what matters to their populations.<br>Connect industry to citizens and the public.	Use DH to offer additional touchpoints to patients.<br><br>Slide step from the hierarchical role of before and support more open space working, and cooperative ways of engaging with patients.<br><br>Use the technology.<br>Need the right people in healthcare settings to support optimal use of technology<br>Offer transparency -record and share their quality and outcomes data.	Trusted voices<br>Translate proven benefits to sources that patients will read.<br><br>Communicate more effectively with the data subjects.<br><br>Use simple language to make the research space feel more transparent/open.<br><br>Work with patients to understand the priority areas.<br><br>Seek out data that can help make breakthroughs in medical research...<br><br>Engage patients from early discovery. Seek support to overcome that discomfort barrier.	Encourage mass adoption and rollout of DH.<br><br>Provide regulation to the 'wild west' - in particular areas such as mental health.<br><br>Help to understand which apps meet recommended standards (data, information).<br><br>Engage with patients, citizens, and healthcare consumers as well as other stakeholders.<br><br>Support an enviornment where healthcare setting speed up implementation of effective data management systems and open and transparent data sharing.	Collate budgets/efforts with other industry and have a much bigger impact in developing something needed/worthwhile (reducing duplication)<br><br>Too fast-moving, need to slow down and take a less commercial approach.<br><br>Try to be more balanced and end user focused.<br><br>Be more transparent.	Responsibility to educate the public/users so that tech is used responsibly<br><br>Be bold and focus on areas that have traditionally not been well remunerated, i.e., advanced diagnostics. Be bold about demanding a fair price, 'not just what a hospital is willing to pay' - but will need to demonstrate

Table 4. Stakeholder Expectations Snapshot. Summary of Interview Findings: Policymakers, regulators

	Patients/ Individuals / Advocates on PO	HCPs or healthcare providers	Researchers/ academia	Policymakers, regulators and payers	Industry	Tech/digital companies
Policymakers, regulators and payers	Provide patient experience data via digital channels.<br><br>Be open to education about the benefits of Digital Health<br><br>Get involved, be patient and contribute - particularly from marginalised groups<br><br>Users of the systems	Support clinical data standards<br><br>Gaining 'digital confidence'<br><br>Understand the benefits DH could offer to workforce<br><br>Dispel myths/discuss fears about litigation and responsibilities (re remote monitoring etc.)<br><br>Also 'users' of the systems.	Need more evidence, from better quality data, that comes from co-design<br><br>Providing ethics of research. Thinking through the guardrails, ethics and challenges of digital and digital tech.<br><br>Leverage digital solutions in order to conduct research ethically.	Overall responsibility to make sure we're doing the right thing. Ethics frameworks.<br><br>Shape and influence frameworks and guidance e.g standards on cybersecurity<br><br>Help data to flow, separate from the applications - needs infrastructure and standards.<br><br>More consistency from policymakers (less political) i.e clear and consistent priorities<br><br>Support a trust mark for DH solutions	Educate about their role - make it clear that they may provide a tool but they don't have access to the data etc.<br><br>Building trust with the public and being very clear<br><br>Think about interoperability and how solution is going to support workforce burden<br><br>Work with academia to develop the evidence base<br><br>Consolidate solutions across brands. Don't overcrowd marketplace and overwhelm patients	Primary role is to create and develop technology to improve patient outcomes<br><br>Be a true partner to the health ecosystem - work with other stakeholders to create something more robust, build trust and navigate for the health system.<br><br>Don't create data in silos

Table 5. Stakeholder Expectations Snapshot. Summary of Interview Findings: Industry.

	Patients/ Individuals/ Advocates on PO	HCPs or healthcare providers	Researchers/ academia	Policymakers, regulators and payers	Industry	Tech/digital companies
Industry	Effective partners in development. Can support regulatory decisions and support better outputs.<br><br>POs are the link between individuals and the developers - but to reach the wider (non-patient population) you need the media and other stakeholders involved<br><br>Patients should advocate harder to ensure GDPR is not used as a barrier to prevent sharing of data for research and better understanding of conditions	Improve digital literacy and trust of digital solutions provided by industry<br><br>For many patients, it's important that **HCPs recommend solutions. HCPs'** role here is for physicians to become trusted partners. How do we build this trust so that they can become the gatekeepers?<br><br>**Support industry to understand what their patients need**<br><br>Translate DH data into clinical decision making<br><br>Support patients to use tools	Bringing the independent, evidence-based view.	Help keep industry focused on the issues that really matter and highlight real areas for change.<br><br>Acceptance of the patient voice into the approval or endorsement process<br><br>Payors (insurers) can encourage take up of DH	Focus on building relationships with patient groups and ensure diversity<br><br>Enablers for patient engagement - have the resources and background/experience<br><br>Still learning and going through a digital transformation	Comply with regulatory frameworks

Table 6. Stakeholder Expectations Snapshot. Summary of Interview Findings: Tech/ Digital Company.

	Patients/ Individuals/ Advocates on PO	HCPs or healthcare providers	Researchers/ academia	Policymakers, regulators and payers	Industry	Tech/digital companies
**Industry**	Take an active role in the adaptation of DH technologies<br><br>Their real-world experience needs to be sought, and acted upon<br><br>POs to advise on where to find good-quality information<br><br>Take a seat at the table<br><br>Hold people accountable, be ethics-oriented and serve the interests of the patient<br><br>Co-creation<br><br>As **citizens,** we also have a role to be informed and be empowered. Being able to question is knowing how to question first.<br><br>Be challenging. Pause and reflect on the value of the proposition and hold sponsors to account.	Advance with technology. Understand what DH can offer and how to engage effectively with it and their patients<br><br>Be advocates for the patients and provide knowledge and incentives to patients to use technology<br><br>Develop the skill competencies to use the technology to improve care<br><br>Ensure involvement in the development process so solutions work well within the healthcare setting.<br><br>Continual learning processes<br><br>Co-creation - providing insights, experiences, learnings, doubts<br><br>Ensuring patients have the tools, the resources to learn to develop their DH literacy<br><br>Create an open and non-judgmental space for patients to ask further questions.	Help to optimise what already exists and reduce duplication of solutions<br><br>Providing theoretical insights<br><br>Identify the effectiveness of best practice<br><br>Engaging with patients directly during their research. Ensure the voice of the end user is captured and broadcast.<br><br>The value of the patient voice should be taught in all university courses about health research.	Create the basis and infrastructure for data sharing<br><br>Commissioners are responsible for bringing about cultural change<br><br>Enforce the safeguarding and regulation of health data<br><br>Enforce compliance from tech companies with standards and regulations<br><br>Keep guidance and policies simple and up to date<br><br>Ensure that solutions reach minority or vulnerable populations<br><br>Foster and facilitate a more rapid uptake of relevant technologies	Engage patients to understand gaps and to create solutions that genuinely benefit and improve outcomes for patients<br><br>Sponsor solutions<br><br>Ensure commitment to solutions that don't dwindle over time<br><br>Be as transparent as possible<br><br>Help more with data protection. For example, inclusion criteria, dimensions.<br><br>Ensure solutions are representative of society.	Look after data security.<br><br>Ensure a good user experience<br><br>Comply with regulatory requirements and to the concept of data safety<br><br>Those developing DH solutions should undergo an ethics and governance course<br><br>Ensure that the technology they are developing is very much fitted with the need and setting of HCPs. Work much more closely with HCPs<br><br>Participate in hackathons or health data challenges, to develop the tools more effectively<br><br>Consider the full ecosystem when developing solutions - do not create data in siloes - needs to integrate into a wider set of data to create meaning for patients
