# Supplementary material for: Digital Health Technologies: Learnings and Perspectives From a Patient Engagement Stakeholder Expectations Matrix Study
Source: J Med Internet Res. 2025 Dec 9;27:e81463. doi: 10.2196/81463 (PMC12728396; doi:10.2196/81463)
Supplement: Multimedia Appendix 3 [file jmir_v27i1e81463_app3.pdf]

This is a Multimedia Appendix to a full manuscript published in the J Med Internet Res. For full copyright and citation information, see http://dx.doi.org/10.2196/jmir.81463

Selected quotes from SEM interviews

**Interview theme**	
**Overall perception of digital health**	*"If there was [consensus on definition of digital health] then the EU, WHO, NIHR, FDA would all accept a single definition. So the powers that be, they themselves come up with what is good for them; what fits with their needs and with their stakeholders' requirements."* ***(Digital/tech company)***
	"Digital health should be an amplification of 'analogue healthcare'. There should be an aspiration to move to a more blended approach to care, which uses digital modalities of whatever type to enhance interactions with the health care system or therapy, where appropriate." ***(HCP/provider)***
	"[Patients] are not always going to base their satisfaction on a clinical outcome, it will be based on various things, starting from how long it took, for example. Even that first conversation with the health system can be part of that experience. Outcomes are not just the clinical outcome; they are broader than just indicators and numbers; it is an experience." ***(HCP/provider)***
	"Everything that is digitised and relates to health could be considered 'digital health' at the widest definition. If you start to categorise, you might end up eliminating some that have potential benefits. On the other hand, if the definition is broad, people may understand the wrong thing about it and include things that are not relevant." ***(Patient/individual/Patient Organisation)***
	"Allows access to a holistic view of a patient's health in the context of them living their daily life." ***(Patient/individual/Patient Organisation)***

	"It could mean anything from a software solution included in a healthcare offering, but it could be a fully digital offering; it could be there's a software component of something that is hardware; it could mean fully remote or could also be that you are doing some exercise digitally whilst you are still in the hospital room." ***(Patient/individual/Patient Organisation)***
	"A patient is not a temporary state. [...] We will be patients; we are patients; everyone is a patient. But the fact that we use the term patient creates a division, as though there are patients and then there is everyone else. It is about messaging and adjusting that division." ***(Tech/digital company)***
	"Digital health is the use of technology to enhance the delivery of health care. When I say the use of technology, it involves the people, the process, and the technology. It's not just about the technology. How do you enable them to be as involved as real partners in their care as possible? Tech now provides opportunities for that that never existed before." ***(Policymaker/regulator/payor)***
**Patient engagement, digital literacy, education, and awareness**	*"I don't think [patient engagement] is sufficiently considered whether you're talking about drugs or digital health, to be honest. So I don't think this is any difference. I think there's still a lot of progress to be made even in the most traditional technologies."* ***(Policymaker/regulator/payer)***
	"When I think about planning my projects and budgeting… If there is a unified methodology, and if this methodology recommends to include focus groups from patients, then it's easier to bring it into your budget than just me saying, I'm the expert. I need this in my budget. I would get this external proof that this way of development could and would be the gold standard. And then you can have a much more informed discussion internally." ***(Industry)***

	"The Scandinavian approach to software development and participatory processes, participatory design, was formalized in law. In the Norwegian Labour Act of 1978, there was a clause saying that when going digital, the beneficiaries or the affected parties should be represented, and they should even have the costs of acquiring new competence to fully participate covered by employers. The rest of Europe is still catching up." ***(Academia)***
	"*As much as we talk about patient centricity, for the pharma industry there's a big fear about regulation and compliance and not interacting directly with patients*" ***(Academia)***
	"[Guidelines/frameworks] actually become quite turgid and meaningless to people when you actually start to write these things down. People want the lived experience - so you need both. I think writing documents that just sit there might appeal to a few people, but they don't appeal to the masses. Documentation supported by real world stories is probably the right way to try and share learning." ***(Policymaker/regulator/payor)***
	"I don't think digital literacy necessarily should block engagement, it just might block the way you engage and develop your communication channels." ***(HCP/provider)***
	"I think the advantage now is that there are no standard processes yet. For drug development, all the processes were very heavily regulated; and it is much more difficult for patients to be part of that process. With digital, because it's new, and everybody understands that it's in their pocket – the patient has an active role to play. There's a different need to interact with patients than before." ***(Patient/individual/Patient Organisation)***
**Digital health in practice**	*"There are a lot of steps, knowledge, expertise and human resource that sit between the digital and the health outcome. With that disclaimer, I would say that we could not run an intensive care unit without digital support. There are also a lot of advantages to using digital support for*

	specific tasks and support in primary and community care – i.e. medication management, information resources etc.” ***(Academia****)*
	“The pandemic really put us through a stress test. And I think people had to learn the hard way about COVID-19, what it is, and vaccinations. So that was successful in a sense that people had to learn. It would be nice if we could leverage that, to navigate that excitement around health and how to learn about health, using digital tools moving forward and outside of the pandemic.” ***(Patient/individual/Patient Organisation)***
	“Pre COVID, it was seen as a niche thing, a thing that people didn't really get to grips with. A lot of people were concerned about the technology or just didn't see how it would make a difference. And what really changed during the COVID pandemic was just an understanding of yes, this could really make a difference. Since, the issue has accelerated, a lot of thinking and a lot of actual implementation of digital health has happened.” ***(Policymaker/regulator/payor)***
	“Our products that we have delivered up to this point have not been digital products and it is not what our infrastructure has been built to deliver. Having that infrastructure that instead allows us to deliver digital products, that is a very large challenge”. ***(Industry)***
	“There are cases where they use [digital health] to completely replace traditional roles of access and that is not a good outcome, because it's not providing an equitable health solution for all, or improving access for the greater good. But if you're using it as part of traditional models of access, and backing it up with great governance processes, in a functioning organization, you've improved access. I think if you can improve access for patients, that's fantastic. ***(HCP/provider)***
**Data management and ownership**	*“I try to stay away from the term ‘ownership’ because it may imply certain risks for patients. The legal definition of ownership implies that it can be changed, meaning you own something today, but you don’t own it tomorrow. This is not the case with data or anything to do with health.*

	You don't own your DNA, it's who you are." ***(Patient/individual/Patient Organisation)***
	"It's difficult to envision that the data will not belong to the medical care team, the health systems or the individual institutions that provide care to the patients when it comes to electronic health data. With patient registries, for example, who owns the data? The patient advocacy group collecting it or the technology vendor that developed the platform? I think I would prefer a patient advocacy groups to actually own the data – can use the data and decide how it is used."<br><br>***(Patient/individual/payor)***
	"Patients are probably the least problematic piece of this. I think it's the system. Whenever you talk to patients, they're very happy to contribute data. There is still an implicit trust that when you consent for your data to be used for health research, that people will use it in the right way. Obviously, there's a big difference between the general public and the individual patients who participate in research." ***(Academia)***
	"Patients often want to donate their data. [General Data Protection Regulation] is doing a bit opposite in a way that boxing it, hiding it, and over protecting it." ***(Policymaker/regulator/payor)***
	"People need to be able to see what is possible when data are allowed to flow," ***(Tech/digital company)***
